# Burden, risk assessment, surveillance and management of SARS-CoV-2 infection in health workers: a scoping review

**DOI:** 10.1186/s40249-020-00756-6

**Published:** 2020-10-07

**Authors:** Federica Calò, Antonio Russo, Clarissa Camaioni, Stefania De Pascalis, Nicola Coppola

**Affiliations:** Department of Mental Health and Public Medicine – Infectious Diseases Unit, University of Campania Luigi Vanvitelli, L. Armanni 5, 80131 Naples, Italy

**Keywords:** Healthcare worker, Health worker, Risk assessment, Surveillance, Management, COVID-19

## Abstract

**Background:**

Health workers (HWs) are at increased risk for severe acute respiratory syndrome-coronavirus-2 (SARS-CoV-2) infection and a possible source of nosocomial transmission clusters. Despite the increased risk, the best surveillance strategy and management of exposed HWs are not yet well known. The aim of this review was to summarize and critically analyze the existing evidence related to this topic in order to support public health strategies aimed at protecting HWs in the hospital setting.

**Main text:**

A comprehensive computerized literature research from 1 January 2020 up to 22 May 2020 was made to identify studies analyzing the burden of infection, risk assessment, surveillance and management of HWs exposed to SARS-CoV-2. Among 1623 citation identified using MEDLINE, Embase, Google Scholar and manual search, we included 43 studies, 14 webpages and 5 ongoing trials.

Health workers have a high risk of acquiring infection while caring for coronavirus disease 2019 (COVID-19) patients. In particular, some types exposures and their duration, as well as the inadequate or non-use of personal protective equipment (PPE) are associated with increased infection risk. Strict infection prevention and control procedures (IPC), adequate training programs on the appropriate use of PPE and close monitoring of HWs with symptom surveillance and testing are essential to significantly reduce the risk. At the moment there is not enough evidence to provide precise indications regarding pre-exposure prophylaxis (PrEP) and post-exposure prophylaxis (PEP).

**Conclusions:**

During the spread of COVID-19 outbreak, numerous published papers investigated the epidemiology, risk assessment and prevention and control of SARS-CoV-2. However, more high-quality studies are needed to provide valid recommendations for better management and for the clinical and microbiological surveillance of healthcare personnel.

## Background

The severe acute respiratory syndrome-coronavirus-2 (SARS-CoV-2), the virus that causes the coronavirus disease (COVID-19), has rapidly worldwide, so on 3 June 2020 approximately 6 348 900 cases of COVID-19 were reported worldwide, with about 380 810 deaths [1].

Current evidence suggests that SARS-CoV-2 is transmitted from person to person through close contact and droplets. People most at risk of acquiring the disease are those who are in contact with or care for patients with COVID-19. This inevitably places health workers (HWs) at a high risk of infection [2–4], citing them as a possible source of nosocomial transmission clusters. In fact, since the pandemic started, healthcare facilities are high-risk places of infection, and some studies report outbreaks in some of these [5–7]. Therefore, knowing the main transmission modes and assessing the risk of HWs are of utmost importance to prevent nosocomial transmission.

As regards the transmission modes of SARS-CoV-2, apart from the known routes such as contact and droplets [8], little evidence is at present reported on aerosol transmission, contaminated environments, the ocular surface and fecal-oral routes as a means of occupational exposure. Considering aerosol transmission, although some studies show the presence of SARS-CoV-2 RNA in aerosols, with lower values in isolated negatively pressurized rooms with high air exchange rate, and higher values in small rooms without ventilation (e.g. patient’s toilet) [9], there is no evidence that SARS-CoV-2 is spread by airborne route [10]. However, it is reported how some procedures produce aerosol with different risks of aerosolization [10]: bronchoscopy, percutaneous tracheostomy and cardiopulmonary resuscitation have an estimated extreme risk of aerosol generation; suctioning, and extubation have a high estimated risk of aerosol generation, while oxygen facemasks, high-flow cannula, non-invasive ventilation and nebulizers have a high to moderate risk [10].

Based on the routes of transmission, current personal protective equipment (PPE) and infection control guidelines [8, 11, 12] have been published and updated giving directives on how to protect the healthcare personnel from infection. In general, isolation gown and gloves are recommended as a contact precaution, and eye protection and surgical masks or filtering face piece-FFP2- (or equivalent) are recommended as a droplet precaution [8, 11, 12]. Airborne precautions, which include the use of FFP3 (or equivalent), are limited for aerosol generating procedures. Furthermore, the guidelines suggest strict procedures for putting on and safely removing PPE [8, 11, 12].

United States Centre for Disease Control (CDC) and European Centre for Disease Prevention and Control (ECDC) guidelines [11, 12] indicates in sequence to putting on, after carefully choose the right PPE, as first the gown, followed by the filtering face piece respirator or facemask, face shield or googles, the gloves. For safely remove the PPE the sequence advised include to put off as first the gloves and the gown, followed by the face shield, the filtering face piece respirator or facemask. Moreover, ECDC define a contact of a COVID-19 case any person, including a HW, who has had contact with a COVID-19 case within a time frame ranging from 48 h before the onset of symptoms of the case to 14 days after the onset of symptoms [13].

As for other HWs, forensics workers are considered at risk of infection, especially during autopsies or managing specimens of COVID-19 patients [14–18]. Generally, PPE used in this setting are similar with some difference. Double surgical gloves interposed with a lyer of cut-proof synthetic mesh gloves, gown fluid resistant or impermeable, waterproof apron, googles or face shield, and filtering face piece respirator or facemask [14]. The guidelines also indicate the correct procedures for sample collection and material disposal [14].

As regards the assessment of risks to HWs, there is a lack of infection surveillance data and unknown or scanty data on the most appropriate way of preventing nosocomial transmission. Therefore, due to the importance of healthcare facilities in the transmission of SARS-CoV-2 worldwide, it is clear that strict infection prevention and control (IPC) procedures, adequate training programs on the appropriate use of PPE and close monitoring of HWs are critical for occupational safety. In addition to being potentially at increased risk of infection with COVID-19, HWs may be responsible for nosocomial outbreaks and may transmit SARS-CoV-2 to vulnerable patients [19, 20].

This scoping review article will focus on the risks of HWs acquiring SARS-CoV-2 infection while carrying out their occupational duties and on the surveillance and management of exposed HWs. The aim was to summarize and critically analyze the existing evidence related to this topic in order to support public health strategies aimed at protecting HWs in the hospital setting.

## Methods

### Search strategy

A comprehensive computerized literature research was made to identify studies analyzing risk assessment, surveillance and management of HWs exposed to SARS-CoV-2 using MEDLINE, Embase, Google Scholar, involving both medical subject heading (MeSH) terminology and relevant keywords for search strings to locate articles that analyzed the infection-control procedures for COVID-19 in healthcare facilities.

The following items were used to search for the studies: “health workers”, “healthcare personnel”, “infection-control”, “pre-exposure prophylaxis”, “post-exposure prophylaxis” “HCW”, “HCP” and “COVID-19”, “SARS-CoV-2”. In addition, the reference lists of all studies meeting the inclusion criteria, of the studies excluded and of the published review articles were manually searched to identify any other study that might merit inclusion. We performed a manually search in CDC, ECDC, World Health Organization (WHO), Clinicaltrials.gov, and European Ministry of Health website in order to include relevant citations.

### Inclusion and exclusion criteria

Due to the rapid spread of the COVID-19 pandemic, the review included studies related to health workers published between 1 January up to 22 May 2020. We included without restrictions guidelines, original article, meta-analysis, letters to editor, editorials, reviews, clinical studies and epidemiological studies, in addition to peer-reviewed publications. Data provided by international organisations and government institutions were also included. All the studies discussing the risk assessment, surveillance and management of HWs exposed to SARS-CoV-2, were included.

Exclusion criteria were a) studies published before 1 January 2020; b) meeting abstract, meeting papers.

### Selection of studies and data extraction

Three researchers (FC, AR and CC) working independently extracted the data according to the inclusion criteria. Disagreements on the inclusion or exclusion of literature were resolved through discussion. The following relevant information was collected from every article selected according to the inclusion criteria: last name of the first author, year and name of journal of publication, country where the population was investigated, study design and topic. Duplicate articles were eliminated.

### Synthesis of results

Based on the main research objectives, articles were classified into one of the following research topics: epidemiology, risk factors and prevention and control, diagnosis and surveillance, management. “Epidemiology” included studies on the epidemic distribution of SARS-CoV-2 in HWs; “risk factors and prevention and control” included studies on risk assessment, transmission patterns and IPC procedures; “diagnosis and surveillance” included studies on microbiological diagnosis and surveillance of HWs exposed to COVID-19; and “management” included studies on pre and post-exposure prophylaxis measures.

## Results and discussion

**Fig. 1:**
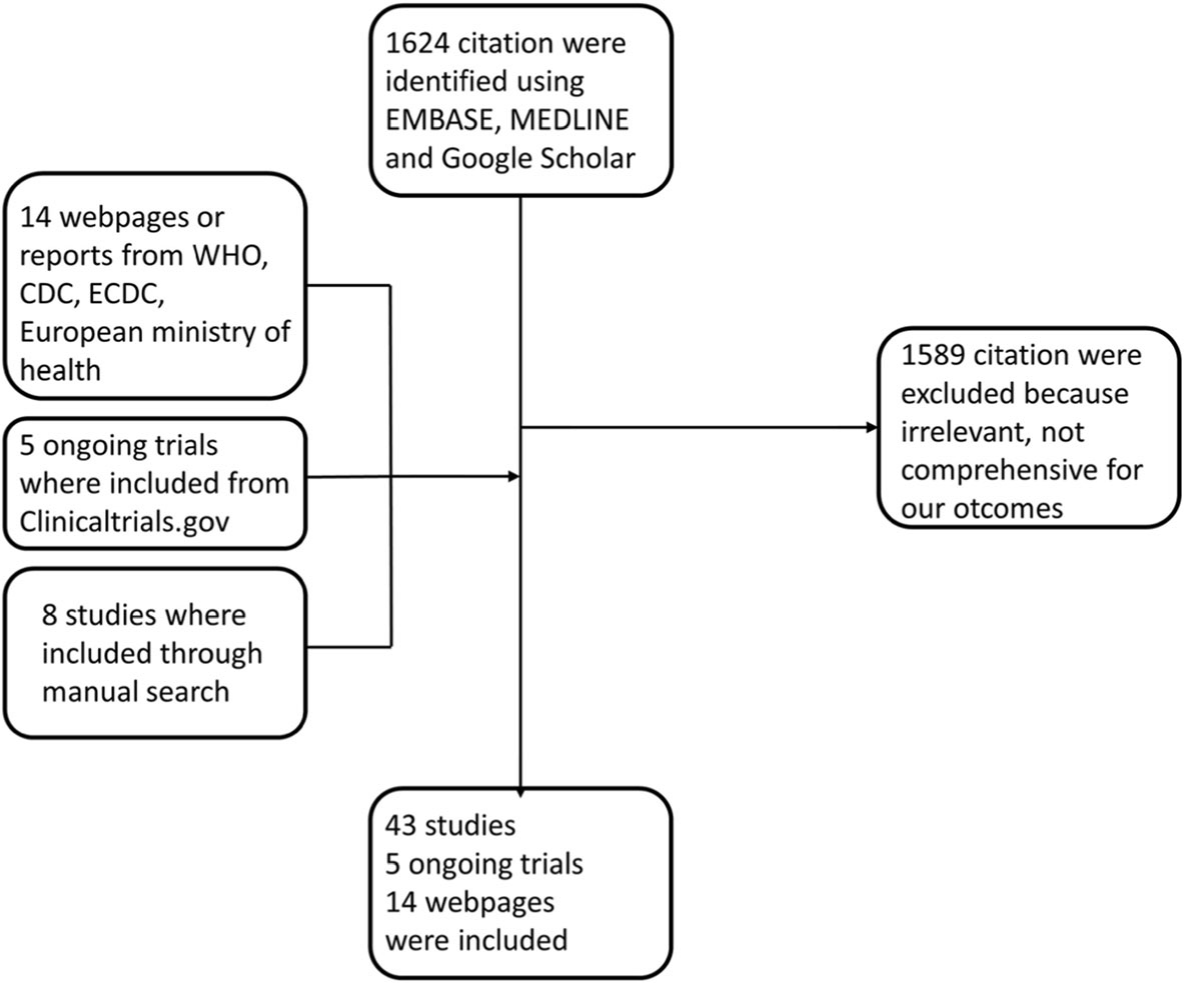
Flow chart of studies selection

The flow diagram (Fig. 1) shows the process of identification and selection of the articles included in the meta-analysis. Using MEDLINE, Embase and Google scholar we identified 1624 citations; of these 43 (8 from manual search) were the most comprehensive for our outcome. From WHO, CDC, ECDC, European ministry of health we selected 14 webpages or reports. We manually searched on Clinicaltrial.gov and included 5 ongoing studies following our outcome.

### Burden of SARS-CoV-2-infection in HWs

In several countries worldwide, the proportion of COVID-19 cases among healthcare professionals was reported as highly variable (Table 1).

**Table 1:** Burden of SARS-CoV-2 infection in health workers (HWs) in different studies

Author, reference number	Country	Type of study	Studies in general population		Studies in HWs		Notes
			Number of COVID-19 patients	Number (%) of HWs COVID-19	Number of HWs tested	Number (%) of HWs COVID-19	
Kang et al., [16]	Korea	National Registry	10 062	241 (2.4)	–	–	Data updated to 5 April 2020
Rivett et al., [21]	United Kingdom	Cohort study	–	–	1032	30 (3)	–
Reusken et al., [22]	Netherlands	Cohort study	–	–	1097	45 (4.1)	–
Kluytmans et al., [23]	Netherlands	Cohort study	–	–	1353	86 (6)	–
Ministry of Health, [17]	Italy	National Registry	228 418	27 101 (11.9)	–	–	Data updated to 22 May 2020
Hunter et al., [24]	United Kingdom	Cohort study	–	–	1654	240 (14)	–
Ministry of Health, [19]	Spain	National Registry	250 287	40 921 (16.3%)	–	–	Data updated to 21 May 2020
Keeley et al., [11]	United Kingdom	Cohort study	–	–	1533	282 (18)	–
Wang et al., [15]	China	Retrospective, single- center case series	138	40 (29)	–	–	–
Folgueira et al., [25]	Spain	Cohort study	–	–	2085	791 (38)	–

- data not reported

Of the all confirmed cases in China, about 3.8% were HWs with the majority of cases (88%) reported from Hubei [26, 27]. Indeed, through 25 February 2020, 3387 SARS-CoV-2 positive HWs were reported in Hubei, at least 18 of whom died [28]. Another report from a single-center case series of 138 hospitalized COVID-19 patients in Wuhan, China, in January 2020 found that 29% (40/138) were HWs infected in hospital [29].

In Italy of the 228 418 confirmed COVID-19 cases, from 11.9% up to 20% were reported in healthcare personnel [22, 25, 27]. In Spain, the latest COVID-19 situation overview from the Ministry of Health showed that among the 250 287 COVID-19 cases, 40 921 (16.3%) were HWs [24]. In the US, the reported cases of COVID-19 among HWs ranged from 3 to 11%, depending on the states with more complete reporting [30]. From an overall 1423 SARS-CoV-2-positive HWs, 55% reported a known contact with a confirmed COVID-19 patient in the 14 days before the illness onset [30].

The different prevalence of SARS-CoV-2 infection in HW in the different countries may be explained by several factors. For example, by the different prevalence of SARS-CoV-2 infection in the general population, the different IPC procedures and positive tracking protocol used. Moreover, different seems to be the prevalence of SARS-CoV-2 infection in HW compared to that observe during severe acute respiratory syndrome (SARS) and Middle East respiratory syndrome coronavirus (MERS) epidemics. In fact, HWs accounted for a large proportion of persons with SARS during the worldwide epidemic of early 2003, accounting for 21% of over 8000 known cases reported from 33 countries in 5 continents [31]. As regards MERS, the majority of cases were registered in Saudi Arabia, the United Arab Emirates and Republic of Korea [32–34]. In Saudi Arabia, 19.1% of all cases of MERS reported from January 2013 to November 2019 were HWs [35]. As of 5 April 2020, the Korea Centers for Disease Control and Prevention (KCDC) reported 241 COVID-19 cases in HWs, 101 infected at work, among a total of 10 062 COVID-19 cases, suggesting a less rate of COVID-19 infection in HWs comparing to that observed for MERS (2.4% vs 21%) [36]. They attribute this lower rate of infection despite an higher reproductive number (*R*_0_) ranging from 2 to 5.7 for SARS-CoV-2 to < 1 for MERS, to a better management of suspected patients and the management of COVID-19, the widely use of PCR test, implementation of contact tracking, the implementation of epidemiological team, the training in good practices and the proper use of PPE including, the practice of wearing mask both in hospital staff and visitors [36]. However, the cases reported among healthcare professionals are not always the expression of occupational exposure. For example, in a study [37] carried out in the Netherlands in nine hospitals from 6 to 8 March 2020, a total of 1097 HWs without a known epidemiological link for professional COVID-19 exposure (close contact with confirmed case) were tested for SARS-CoV-2, of whom 45 (4.1%) were found positive. A Spanish study [38] conducted in a large public hospital in March 2020 found a rate of SARS-CoV-2 infection in 38% of HWs tested and 11.6% in all hospital workers; interestingly, no significant difference in the SARS-CoV-2 infection was observed between HWs from high-risk areas involved in close contact with COVID-19 patients and clerical, administrative or laboratory personnel without direct contact with patients. Similarly, the Newcastle upon Tyne Hospitals NHS Foundation Trust screened staff with compatible symptoms (i.e., new continuous cough or fever) and found no difference in SARS-CoV-2 positivity rates between non-clinical staff (e.g., clerical, administrative, information technology, secretarial, etc.) and frontline staff (e.g., nurses, doctors, allied healthcare professionals, porters, etc.) [39]. These results seem to suggest that several infections of HWs may be related to household or community contacts during the early phase of local spread and that the current isolation protocols and PPE appear sufficient to prevent high levels of nosocomial transmission to frontline staff [39].

### Risk assessment of HWs exposed to SARS-CoV-2

The assessment of the risk is mandatory to identify the correct strategies to avoid SARS-CoV-2 infection in HWs. In the analysis of risk the type of contact, the risks related to environmental contamination and adherence to IPC measurements should be considered. Based on the level of exposure and the use of adequate PPE, a high-risk exposure was defined as a HW providing care to a COVID-19 case, or laboratory workers handling specimens from a COVID-19 case without the recommended PPE or with a possible breach of PPE. A low-risk exposure was defined as a HW wearing the recommended PPE. Although the correct use of PPE was not associated with SARS-CoV-2, in real-word, a deviation of protocol may be occurred with a possible infection. The exposure category, the risk assessment and the recommended monitoring are summarized in Table 2.

**Table 2:** Risk assessment and recommended monitoring of health workers (HWs) exposed to SARS-CoV-2

Exposure category	Circumstances	Recommended monitoring
*High-risk exposure*	HW who has assisted a COVID-19 case and performed procedures that generate aerosols or manipulated biological samples during which direct exposure of the skin or mucous membranes occurred without adequate PPE.	Stop all health care interaction with patients and get tested for COVID-19; Quarantine and daily self-monitoring of temperature and respiratory symptoms for 14 days after the last day of exposure to a COVID-19 patient.
*Medium-risk exposure*	HW who had prolonged close contact with patients with COVID-19 not equipped with the indicated PPE without direct exposure to the patient’s biological materials or in the event of non-compliance with the procedures indicated.	Daily self-monitoring of temperature and respiratory symptoms for 14 days after the last day of exposure to a COVID-19 patient and weekly active surveillance.
*Low-risk exposures*	HW who has assisted the case or manipulated biological samples, with the indicated PPE, and without accidents or episodes discordant with the indicated procedures.	Daily self-monitor of temperature and respiratory symptoms for 14 days after the last day of exposure to a COVID-19 patient and weekly active surveillance.

Risks associated with environmental contamination and the use of PPE are shown in Table 3. However, we underline that if all the required procedures, such as isolation possibly in negative pressure rooms, correct use of PPE, correct hygiene, are followed, the contamination of the hospital facilities would not be happened. However, knowing the extent of viral environmental contamination in COVID-19 wards is critical for improving safety practices and risk assessment for medical staff. Guo et al. [40] in a study conducted in Wuhan, China, analyzed air and surface samples of intensive care units (ICU) and general wards (GW). The authors found the rate of SARS-CoV-2 positivity much higher for the ICU than for the GW (43.5% vs 7.9%); in particular, the rate of positivity was relatively high for floor swab samples (70% vs 15.4%), suggesting that medical staff should perform stricter protective measures of infection control, such as disinfection of shoe soles before walking out of wards containing COVID-19 patients. The same authors assessed the risk for aerosol transmission of SARS-CoV-2 collecting air in the isolation ward of the ICU (12 air supplies and 16 air discharges per hour) and GW (8 air supplies and 12 air discharges per hour) and obtained positive test results for 35% of ICU samples and 12.5% of GW samples.

**Table 3:** Evidences of environmental and PPE contamination and adherence to IPC procedures for SARS-CoV-2-infection

Author	Country	Risk assessment		
		Environmental contamination of SARS-CoV-2 (%)	PPE contamination of SARS-CoV-2 (%)	Adherence to IPC procedures or other risks (associated to SARS-CoV-2 infection)
Zhen-Dong et al., [31]	China	• floor (70) • computer mouse (75) • trash can (60); • sickbed handrail (42.9)	• face shield or medical mask (0) • sleeve cuff (16.7) • gloves (25) • shoe sole (50) • patients mask (40)	
Ye et al., [33]	China	• self-service printer (patient only) (20) • table top/keyboard (16.8) • doorknob (16) • telephone (12.5) • medical equipment (not PPE) (12.5) • wall/floor (5.6)	• hand sanitizer dispenser (20.3) • gloves (15.4) • eye protection or facial shield (1.7)	
Ong et al., [33]	Singapore		• One day HW’s PPE sampling (0)	
Ran et al., [34]	China			• Suboptimal hand washing before (RR: 3.10, 95% *CI*: 1.43–6.73) or after (RR: 2.82, 95% *CI*: 1.11–7.18) patient contact • Improper PPE use (RR: 2.82, 95% *CI*: 1.11–7.18) • Work in a high risk versus general department (RR: 2.13,95% *CI*: 1.45–3.95) • Longer work hours (log-rank *P* = 0.02)
Liu et al., [35]	China			• Close direct contact (within 1 m) with COVID-19 patients • Average number of 12 contacts (range: 7–16) • Average cumulative contact time of two hours (range: 1.5–2.7)
Bartoszko et al., [37]	–			• No differences in rate of infection between medical mask and N95 respirators
Ng et al., [38]	Singapore			• No differences in rate of infection between surgical mask and N95 respirators
Jin et al., [39]	China			• 84.5% of HWs thought they were infected in working environment hospital^a^ • 41.8% of HWs reported that their infection was related to not maintaining protective equipment and not utilizing common equipment (masks and gloves) ^a^ • 4.9% of HWs thought they were infected in daily life or community environment^a^ • 1% of HWs thought that their infection was due to the laboratory environments^a^

*HWs* health workers, *IPC* Infection prevention control, *PPE* personal protective equipment, *RR* relative risk, *OR* odds ratio; *CI* confidene interval.

^a^self-administered questionnaire

In a study performed in healthcare facilities, the most contaminated objects were self-service printers (20.0%), desktops/keyboards (16.8%) and doorknobs (16.0%); both hand sanitizer dispensers (20.3%) and gloves (15.4%) were the most contaminated PPE [23]. On the contrary, in the analysis of 90 samples of PPE from 30 HWs, Ong et al. [41] showed that none was SARS-CoV-2 positive. However, it should be underscored that the time spent in the patient’s room was very little, a median of 6 min (IQR: 5–10): 8 min for medical doctors, 7 min for nurses, and 3 min for cleaning staff.

Other factors to consider in risk assessment of HWs attending SARS-CoV-2 subjects include the duration of exposure, clinical symptoms of the patient (e.g., coughing likely increases exposure risk), whether the patient was wearing a facemask (which can efficiently block respiratory secretions from contaminating others and the environment), whether an aerosol generating procedure was performed and the type of PPE used by HWs. A study evaluating risk factors for COVID-19 in 72 exposed HWs in Wuhan, China who had acute symptoms identified as the risk factors working in a high risk versus general department (relative risk [RR]: 2.13, 95% *CI*: 1.45–3.95), suboptimal hand washing before or after patient contact (RR: 3.10, 95% *CI*: 1.43–6.73; and RR: 2.82, 95% *CI*:1.11–7.18, respectively), longer working hours (log-rank *P* = 0.02), and improper PPE use (RR: 2.82, 95% *CI*: 1.11–7.18]) [42]. Min et al. [21] in a study on 30 SARS-CoV-2-positive HWs in China described all cases having a history of close direct contact (within 1 m) with COVID-19 patients, with an average number of 12 contacts (range: 7–16), and the average cumulative contact time of two hours (range: 1.5–2.7).

Finally, to prevent SARS-CoV-2 infection in HWs, the use of appropriate PPE, including an N95 respirator and isolation gown, has been emphasized, but no difference in avoiding infection was observed between the use of medical mask and FFP2 or N95 mask. In fact, the results of a meta-analysis [43] highlighted that, compared with N95, the use of medical masks did not increase laboratory-confirmed viral respiratory infection, including coronaviruses (*OR*: 1.06; 95% *CI*: 0.90–1.25; *I*^2^ = 0%;) or clinical respiratory illness (*OR*: 1.49; 95% *CI*: 0.98–2.28; *I*^2^ = 78%). Only one trial evaluated coronaviruses separately and found no difference between the two groups (*P* = 0.49) [43]. A study conducted in Singapore on 41 HWs exposed to aerosol-generating medical procedures for at least 10 min within 2 m of the undiagnosed COVID-19 patient found no case of SARS-CoV-2 infection in the 35 HWs who wore surgical masks and in the six who wore N95 respirators [44].

Concluding on this point, for risk assessment, healthcare facilities should use questionnaires to proposed healthcare personnel [2] to investigate the type of healthcare personnel (e.g. medical doctor, registered nurse, etc.), type of healthcare facility (e.g. medical unit, ICU, etc.), type of contact (face to face/within 1 m or greater distance) and type of procedure performed on the confirmed COVID-19 patient (e.g. tracheal intubation, nebulizer treatment, tracheotomy, bronchoscopy, etc.). Furthermore, these questionnaires would be a useful tool to verify compliance with IPC such as the correct use of PPE in accordance with the type of procedure and the possible accident with body fluid/respiratory secretions during the care of COVID-19 patients. For example, in Wuhan University out of 103 COVID-19 HWs who completed a validated questionnaire on the causes of infection, 43 (41.8%) reported that their infection was related to not maintaining protective equipment and not utilizing common equipment (masks and gloves) when at close contact with infected cases. In particular, swabs of throat collection and physical examination were perceived as the procedures most likely causing their infection [45].

### Surveillance strategy of HWs exposed to SARS-CoV-2

**Fig. 2:**
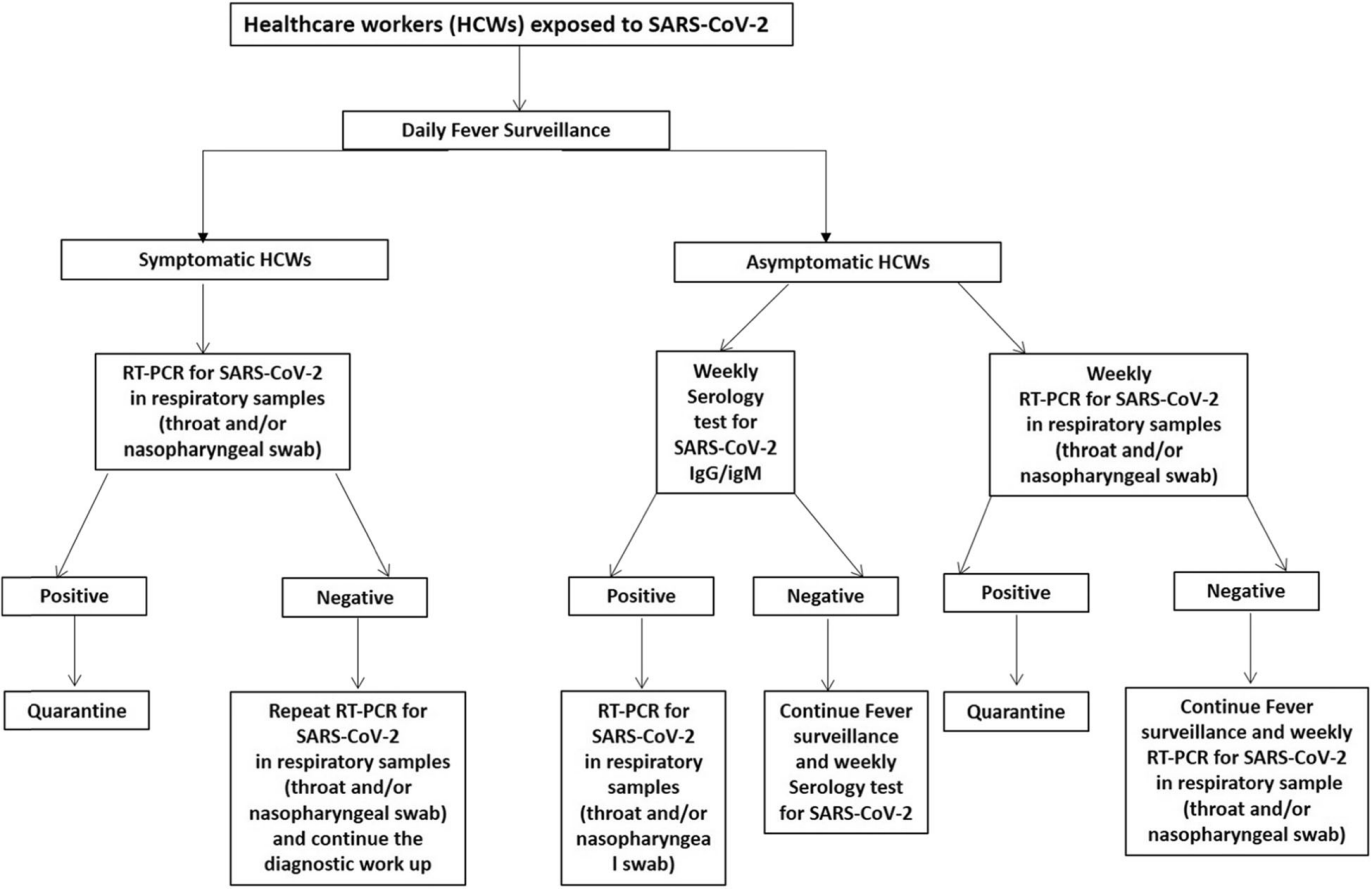
Flow-chart for healthcare surveillance of HWs exposed to SARS-CoV-2hb

The surveillance of HWs exposed to SARS-CoV-2 is aimed at an early identification of the infected subjects to avoid the spread of a nosocomial outbreak. There are two main surveillance strategies for HWs, one passive or clinical and the other active or microbiological. The flow chart on healthcare surveillance strategies of HWs exposed to SARS-CoV-2 is shown in Fig. 2.

#### Passive or clinical surveillance strategy

A passive strategy foresees that all HWs self-assess for fever and/or a defined set of newly present symptoms suggestive of COVID-19. Exposed staff need to do active twice-daily fever surveillance for at least 14 days after the last exposure to the source case. If the HW complains of symptoms they should be sent home immediately after testing. However, the need to monitor also the mild symptoms of COVID-19 (myalgia, coughing and/or sore throat and/or common cold with or without fever) should be stressed. In fact, among the 14 SARS-CoV-2-positive HWs due to exposure to two index patients in the neurosurgery ward in Wuhan, China, the main symptoms were myalgia or fatigue (100%), fever (86%) and a dry cough (71%) [46].

The results from staff testing at Sheffield Teaching Hospitals in the UK between 16 and 29 March 2020 showed that of 1533 HWs with symptoms suggestive of SARS-CoV-2, 282 (18%) were positive [19]. In Germany, of 1353 HWs with fever or respiratory symptoms, 86 (6%) were SARS-CoV-2 positive; of these only 3% reported having been exposed to an inpatient known with COVID-19 prior to the onset of symptoms [47].

This strategy is simple and cost-effective. However, it has several disadvantages: the adherence is highly dependent on HW motivation and appropriate self-assessment of the risk; moreover, the asymptomatic and pre-symptomatic HWs are a potential source of infection not identified by the passive surveillance strategy [48]. Regarding this point, we underscore that SARS-CoV-2 infection may lead to a wide range of clinical presentations, from an asymptomatic or pauci-symptomatic form, which may be present in up to 85%, to a severe acute respiratory syndrome [49]. In a study of COVID-19 symptomatic and asymptomatic infection in a skilled nursing facility in Washington, USA, more than half (56%) of the residents with positive test results were asymptomatic at the time of testing and most likely contributed to transmission [7]. Another study carried out in a large UK teaching hospital found that 3% of HWs tested positive for SARS-CoV-2 among 1032 asymptomatic HWs screened by real-time reverse transcription polymerase chain reaction (RT-PCR) [50].

#### Active or microbiological surveillance strategy

An active, or microbiological surveillance strategy foresees that all HWs be routinely tested for SARS-CoV-2 infection. The main diagnostic tools for surveillance in HWs exposed to COVID-19 are essentially real time RT-PCR in respiratory samples (throat and/or nasopharyngeal swab) and serology (point-of-care or laboratory-based) as surrogate for immunity for SARS-CoV-2.

RT-PCR is particularly useful to detect current infection with SARS-CoV-2. A recent meta-analysis performed by Kim et al. [51] considering 19 studies showed a pooled sensitivity of 89% (95% *CI*: 81–94%; *I*^2^ = 90%), while the positive predictive value (PPV) ranged from 47.3% to 98.3% and the negative predictive value (NPV) ranged from 93.4 to 99.9%. Although RT-PCR represents a cornerstone for SARS-CoV-2 laboratory diagnosis, several limitations have been observed: inadequate sample collection or conservation or transportation, expensive test that requires qualified personnel and medium turnaround time.

Several serological immunoassays have been developed for the detection of SARS-CoV-2 viral proteins and antibodies in serum or plasma samples. The most widely used commercial tests are based on rapid lateral flow immunoassay (LFIA), automated chemiluminescence immunoassay (CLIA), enzyme-linked immune assay (ELISA) and rapid IgM/IgG tests, mainly based on immunoassay technology that provides results in 10–15 min [52]. The advantages of serological tests are that they are not very expensive, have easy collection sampling, use widely used techniques for all serological tests and do not need infrastructure, with rapid results for rapid serological tests. Thus, the rapid serological tests may be considered in active surveillance of HWs as an adding value tool. However, antibody responses to infection take days to weeks to be reliably detectable [53]. Therefore, negative results would not exclude SARS-CoV-2 infection, particularly among those with recent exposure to the virus [54]. The sensitivity of the rapid IgM-IgG combined antibody test varies in different studies [55, 56]. Li Z et al. [55] collected blood samples from 397 PCR confirmed COVID-19 patients and 128 negative patients at eight different clinical sites and found an overall testing sensitivity of 88.7% and specificity of 90.6%. Instead, in a real-life study [56] performed in an emergency room department of a tertiary hospital in northern Italy on 110 subjects (30 healthy volunteers, 30 COVID-19-positive patients and 50 patients at their first access to an emergency room department with fever and respiratory syndrome), Cassaniti et al. found that the sensitivity of the rapid test was 18.4%, specificity 91.7%, while NPV was 26.2% and PPV was 87.5% in the patients enrolled [56].

In conclusion, owing to the great variability of the rates of sensitivity of the rapid serological tests they should be used with caution for screening HWs exposed to SARS-CoV-2, and RT-PCR, although expensive and difficult-to-repeat, remains the gold standard in active surveillance.

### Pre-exposure and post-exposure management of HWs exposed to SARS-CoV-2

Currently, while active research on vaccine development is ongoing and although several drugs have been proposed as treatment regimens, there is no validated recommendation for pre- or post-exposure prevention of SARS-CoV-2 infection among HWs [57], and the data on this topic prevalently come from the outbreak of SARS in 2003 and MERS in 2012. A study conducted in Republic of Korea [58] highlighted the efficacy of post-exposure prophylaxis (PEP) of ribavirin and lopinavir/ritonavir, given from 1 to 3 days after the last exposure until day 14, for 22 HWs exposed to MERS-CoV patients: no instance of MERS-CoV infection was detected in these subjects. While, 6 out of 21 HWs retrospectively enrolled in another 4 hospitals, exposed but without PEP, developed MERS-CoV infection.

As regards SARS-CoV-2, in vitro studies showed effectiveness of chloroquine (CQ) at entry and post entry level, suggesting its possible prophylactic activity also for this infection [59]. A systematic review in March 2020, considering three pre-clinical in vitro studies, showed that CQ and hydroxychloroquine (HCQ) have prophylactic effects against SARS-Cov2, but, actually, despite pre-clinical results being promising, in the absence of clinical studies to support the efficacy of CQ or HCQ in preventing COVID-19 CQ and HCQ are not indicate in this setting [60, 61]. In fact, several trials [62–64] are currently underway on the effectiveness of chloroquine as a pre-exposure prophylaxis (PrEP). For example, EPICOS [65] is a randomized clinical trial on HWs in Spain that aims to assess the efficacy of a daily single dose of tenofovir/emtricitabile (TDF/FTC), or HCQ, or TDF/FTC plus HCQ versus placebo as PrEP in 4000 HWs (NCT04334928). A double-blinded, randomized placebo-controlled trial [66] is currently being conducted on 374 HWs in New York to determine whether PrEP, with a daily oral intake of 400 mg HCQ, reduces the incidence of COVID-19 infection (NCT04352946).

Regarding the use of HCQ as PPE in a Korean study, it was administered to 189 patients and 22 HWs (who had initially tested negative for COVID) after exposure to a positive subject: none of the patients or HWs developed SARS-CoV-2-infection. However, in the absence of a control group these data cannot be considered conclusive on the effectiveness of HCQ as a PEP agent for COVID-19 [67]. However, we underline that recently the use of CQ and HCQ was banned by the WHO also in the therapeutic setting of patients with COVID-19.

## Conclusions

This scoping review summarizes the evidence on the burden, risk assessment, surveillance and management of HWs exposed to SARS-CoV-2.

HWs are at a high risk of acquiring infection while caring for COVID-19 patients, due to long working hours, physical and psychological distress, inadequate training and shortage of personal protective equipment. Knowing the risk assessment of the various procedures and environmental contamination is of the utmost importance to implement the appropriate infection control measures. Surveillance is recommended for all HWs who are exposed to patients meeting the case definition for SARS-CoV-2, with the aim of identifying the occurrence of compatible symptoms and for infection control purposes to further limit in-hospital transmission clinical surveillance with fever/symptom assessment may lack information from asymptomatic/presymptomatic HWs. For this reason, it is reasonable that all exposed HWs are subjected, in addition to clinical monitoring, to microbiological surveillance using a swab or serological test, evaluating the pros and cons of the different methods according to the different settings. There are currently no robust data to give precise indications on PrEP and PPE.

However, this scoping review has some limitations: firstly, with new data being published on a daily basis, this review can only provide results up to 22 May 2020; moreover, publication bias may be not excluded. Although, due to the novelty of the epidemic, it cannot reflect the entire body of research on risk assessment and management of HWs exposed to COVID-19 worldwide, it will provide some evidences for future study.

## Data Availability

All data generated or analyzed supporting the findings of this article are included within the article**.**
